# Identification of Bacterial Community Composition in Freshwater Aquaculture System Farming of *Litopenaeus vannamei* Reveals Distinct Temperature-Driven Patterns

**DOI:** 10.3390/ijms150813663

**Published:** 2014-08-07

**Authors:** Yuyi Tang, Peiying Tao, Jianguo Tan, Haizhen Mu, Li Peng, Dandan Yang, Shilu Tong, Lanming Chen

**Affiliations:** 1 Key Laboratory of Quality and Safety Risk Assessment for Aquatic Products on Storage and Preservation (Shanghai), China Ministry of Agriculture, College of Food Science and Technology, Shanghai Ocean University, 999 Hu Cheng Huan Road, Shanghai 201306, China; E-Mails: m110250462@st.shou.edu.cn (Y.T.); tpy2011812@sina.com (P.T.); 2 Shanghai Key Laboratory of Meteorology and Health, 951 Jinxiu Road, Shanghai 200135, China; E-Mails: jianguot@21cn.com (J.T.); muhz@climate.sh.cn (H.M.); phyllis_pl@163.com (L.P.); ydd_danfer@hotmail.com (D.Y.); 3 School of Public Health, Institute of Health and Biomedical Innovation, Queensland University of Technology, Brisbane, Australia Kelvin Grove, QLD 4059, Australia; E-Mail: s.tong@qut.edu.au

**Keywords:** aquaculture, *Litopenaeus vannamei*, bacterial community, temperature, pathogen

## Abstract

Change in temperature is often a major environmental factor in triggering waterborne disease outbreaks. Previous research has revealed temporal and spatial patterns of bacterial population in several aquatic ecosystems. To date, very little information is available on aquaculture environment. Here, we assessed environmental temperature effects on bacterial community composition in freshwater aquaculture system farming of *Litopenaeus vannamei* (FASFL). Water samples were collected over a one-year period, and aquatic bacteria were characterized by polymerase chain reaction-denaturing gradient gel electrophoresis (PCR-DGGE) and 16S rDNA pyrosequencing. Resulting DGGE fingerprints revealed a specific and dynamic bacterial population structure with considerable variation over the seasonal change, suggesting that environmental temperature was a key driver of bacterial population in the FASFL. Pyrosequencing data further demonstrated substantial difference in bacterial community composition between the water at higher (WHT) and at lower (WLT) temperatures in the FASFL. *Actinobacteria*, *Proteobacteria* and *Bacteroidetes* were the highest abundant phyla in the FASFL, however, a large number of unclassified bacteria contributed the most to the observed variation in phylogenetic diversity. The WHT harbored remarkably higher diversity and richness in bacterial composition at genus and species levels when compared to the WLT. Some potential pathogenenic species were identified in both WHT and WLT, providing data in support of aquatic animal health management in the aquaculture industry.

## 1. Introduction

The Pacific white shrimp *Litopenaeus vannamei* is the most widely cultured and productive alien crustacean worldwide [1]. It is native to the western Pacific coast of Latin America, and introduced commercially since 1996 into China and several countries in Asia [2]. In China, the yearly estimated *L*. *vannamei* production was over one million tons during the past several years [1]. The freshwater culture of *L*. *vannamei* has proven even more successful than brackish water culture conditions [2], which play very important roles in the shrimp production in the southeast littoral provinces in China. Along with the fast growing shrimp-production industry, however, aquatic animal diseases caused by waterborne pathogens have also rapidly increased, which led to huge economic losses in the past decades [3]. Previous culture-based studies have revealed that *Vibrionaceae*-related organisms, being virtually ubiquitous in aquatic environments [4], were the major pathogens causing disease outbreaks and mortality of early stage hatchery-reared shrimps including *L*. *vannamei* [5,6,7,8,9]. It is well known that some pathogenic *Vibrios* are also serious human foodborne pathogens causing worldwide cholera epidemics and diarrheal disease [10]. Recently, *Streptococcosis* in farmed *L*. *vannamei* was reported as a new emerging bacterial disease of penaeid shrimp [11]. To elucidate the mechanism underlying the emergence and resurgence of the waterborne pathogens, a complete understanding of the composition and dynamics of microbial population in aquaculture ecosystems is required.

Recent significant development of metagenomic techniques has allowed for culture-independent genomic fingerprinting of bacterial communities in the aquaculture environment [12]. Using the DGGE technique, bacterial community structures have been investigated in various aquaculture settings in different parts of the world, e.g., culturing the tropical rock lobster (*Panulirus ornatus*) in Australia [13], the Pacific white shrimp (*L*. *vannamei*) in the USA [14], the Asian tiger shrimp (*Litopenaeus monodon*) in Thailand [15], the Atlantic cod (*Gadus morhua* L.) in Norway [16], the grass carp (*Ctenopharyngodon idellus*) in Zhangjiang, China [17], as well as the shrimp (*Penaeus vannamei*, *Penacus orientalis*), abalone (*Haliotis diversicolor*) and reef cod (*Epinephelus diacanthus*) in coastal mariculture ponds in Southeast China [18]. These studies have proposed strong links between environmental variables (e.g., season, water flow rate and aeration) and bacterial population structures in the aquaculture niches. Compared to the DGGE and traditional Sanger sequencing of 16S rDNA clone libraries [19], the developed second-generation sequencing techniques significantly improved the researcher’s ability to achieve a more definitive phylogenetic-based description of microbial communities in aquatic ecosystems, e.g., ocean [20], deep sea [21], river [22], spring [23], drinking water [24] and wastewater [25]. Previous studies using the approaches have revealed temporal [26,27,28] and spatial [29] patterns of bacterial communities in marine, lake and river environments. Nevertheless, very little information is available to date for microbial community dynamics in aquaculture ecosystems, despite their great significance in economy and human health. In this study, we combined PCR-DGGE and 454-pyrosequencing techniques to evaluate the influence of environmental temperature change on the structure, composition and diversity of bacterial population in the FASFL in Shanghai, one of the major shrimp production regions in China. Our data uncovered a temperature-driven substantial shift in bacterial population in the FASFL. The information will facilitate better understanding of possible molecular mechanisms underlying waterborne disease outbreaks and seasonal change.

## 2. Results and Discussion

### 2.1. DGGE-Based Bacterial Population Profiles in the FASFL

**Figure 1:**
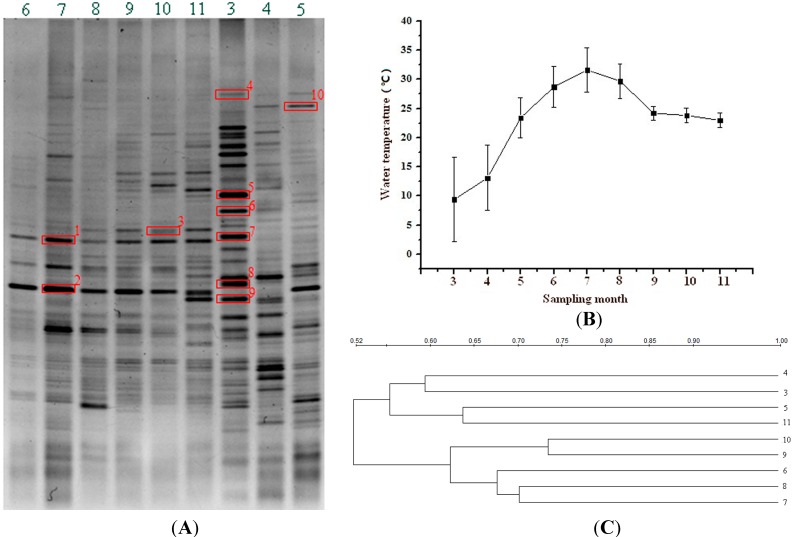
PCR-DGGE analysis of bacterial community structures derived from the water samples in the FASFL in different seasons. On the DGGE gel (**A**), Lane 6 to 11 and 3 to 5 represent the water samples collected from June to November in 2012, and from March to May in 2013, respectively. The DNA bands marked with red boxes on the DGGE gel (**A**) were individually excised and subjected for DNA sequencing. The numbers on the horizontal axis of the temperature curve (**B**) and clustering profile (**C**) represent the sampling months as correspondingly shown in the DGGE gel (**A**).

To gain an insight into the possible influence of temperature on bacterial population in the FASFL, surface water samples were collected over a one-year period (see the Section 3), and aquatic bacteria were characterized by PCR amplification of the V3 variable region of the bacterial 16S rRNA gene. This analysis yielded clear PCR products from all the water samples (Figure not shown). Based on the amplicons, DGGE fingerprints were obtained (Figure 1A), which strongly suggested that the FASFL harbored a complex and specific bacterial community structure. Considerable variation in bacterial composition was observed across the water temperature change (Figure 1B). Cluster analysis of the DGGE fingerprints revealed three distinct groups, designated Group I, II and III (Figure 1C). Group I contained the samples collected in the summer and early autumn (June to October 2012), whereas Group III was comprised of the samples in the early spring (March to April 2013). The late autumn and late spring samples fell into Group II (November 2012, May 2013), showing mosaic fingerprints of the former two Groups. The similarity among the samples was observed in the range from 52% to 74% (Figure 1C), indicating significant difference of intergroup bacterial composition. The data provided the first example of a dynamic bacterial population driven by water temperature in aquaculture environment. Previous studies also revealed distinct seasonal patterns of bacterial population in other freshwater habitats (e.g., river and lake) by PCR-DGGE analysis [27,28].

Selected dominant and distinct bands on the DGGE gel were subsequently sequenced and analyzed (Figure 1A), and revealed phylogenetic diversity of bacterial population in the FASFL across different sampling times (Table 1). The DGGE fingerprints of Group I samples were dominated by three major bacterial bands: one (band 1) was affiliated closely with an uncultured *Rickettsiales* bacterium clone FWB6C1-73; one (band 2) with an uncultured *Micrococcineae* bacterium clone D7N78; the other (band 3) with an uncultured *Methylophilus* sp. clone JA127_2010-09-15 and *Sphingomonas oligophenolica* strain R2A-AUG-EA-11. Interestingly, these bacteria appeared highly sensitive to lower temperature since none of any corresponding bands was observed in Group III patterns, suggesting their thermophilic property. Along with the dropping temperature, Group III fingerprints again changed largely where several bands were dominant in the profile. Almost all of these (band 4–9) were affiliated closely with uncultured bacterium clone sequences belonging to *Bacteroidetes*, *Actinobacteria* and *Firmicutes* (Table 1). These bacteria were absent from Group I samples, the majority of which only occurred in the sample collected in March 2013 with the lowest water temperature, displaying their psychrotolerent feature. In addition, one of the dominant sequences retrieved from Group II patterns (band 10) showed 100% identity to an uncultured *Intrasporangiaceae bacterium* clone M7N57 belong to *Actinobacteria* (Table 1).

**Table 1:** Phylogenetic identity of dominant bands on the DGGE profile.

Band	Clustering Group	Length (bp)		Closest Relative and Database Accession Number	Identity (%)	Taxonomic Description
B1	III	169		Uncultured *Rickettsiales* bacterium clone FWB6C1-73, KF583165.1	100%	α*-Proteobacteria*
B2	III	174		Uncultured *Micrococcineae* bacterium clone D7N78, KC006224.1	100%	*Actinobacteria*
B3	III	194	Uncultured *Methylophilus* sp. clone JA127_2010-09-15, JN866934.1		100%	β*-Proteobacteria*
		169	*Sphingomonas oligophenolica* strain R2A-AUG-EA-11, JX237432.1, KC836618.1		100%	α*-Proteobacteria*
B4	I	194	*Pediococcus ethanolidurans* strain RU12-4		100%	*Firmicutes*
B5	I	189	Uncultured *Bacteroidetes* bacterium, FR647662.1		100%	*Bacteroidetes*
		189	Uncultured *Cryomorphaceae* bacterium clone Jab PL2W2H12, HM486317.1		100%	*Bacteroidetes*
B6	I	174	*Actinobacterium* MS-B-64, FJ460153.1		100%	*Actinobacteria*
B7	I	189	Uncultured *Bacteroidetes* bacterium clone XSLJ052, KC246401.1		100%	*Bacteroidetes*
B8	I	174	Uncultured *actinobacterium* clone FF1G3, EU117678.1		100%	*Actinobacteria*
		174	Uncultured *actinobacterium* clone B12-88, JN371245.1		100%	*Actinobacteria*
		174	*Actinobacterium* SCGC AAA043-A09, HQ663377.1		100%	*Actinobacteria*
B9	I	174	Uncultured *Micrococcineae* bacterium clone D7N78, KC006224.1		100%	*Actinobacteria*
B10	II	174	Uncultured *Intrasporangiaceae bacterium* clone M7N57, KC006381.1		100%	*Actinobacteria*

### 2.2. Environmental Temperature and Bacterial Population in the FASFL

**Figure 2:**
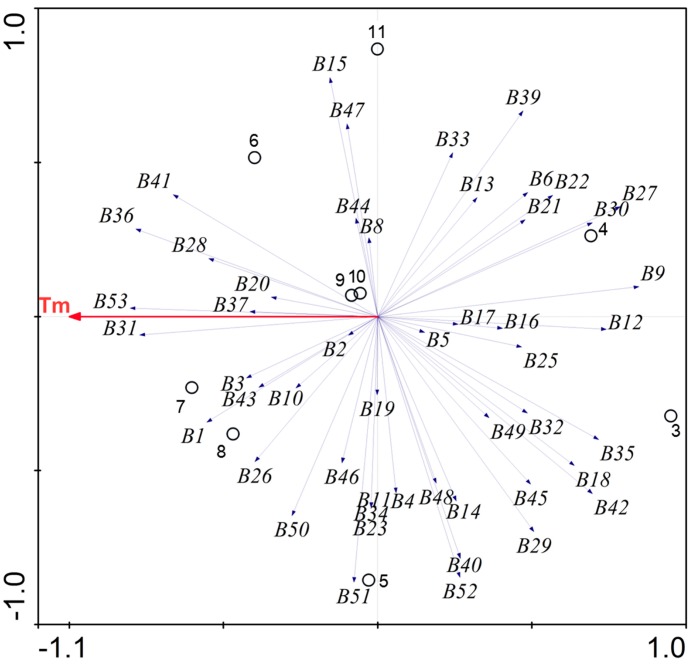
The relationship between environmental variable and bacterial community composition derived from different water samples in the FASFL. DGGE banding scores were plotted using the program CANOCO (Version 4.5). Tm represents the environmental variable of the water temperature. The open closed circles and numbers 3 to 11 indicate different sampling months as described in Figure 1, while the numbers B1 to B52 represent the DNA bands on the PCR-DGGE gel analyzed by the CANOCO, respectively.

To assess the relationship between water temperature and bacterial composition in the FASFL, we did redundancy analysis (RDA) of the DGGE data. As presented in Figure 2, the resulting RDA plot clearly indicated that temperature was positively and closely associated with the bacterial population retrieved from the WHT (≥30 °C). In contrast, a negative correlation was observed for the bacterial community of the WLT (≤20 °C), which located in a completely opposite direction to the temperature variable in the plot. This result was consistent with those of the cluster analysis and band sequencing. Further analysis of similarity (ANOSIM) generated the *R* value of 0.75 (*p <* 0.05) between the WHT and WLT, strongly suggesting a significant separation of the bacterial communities. Thus, the WHT and WLT were subjected for deeper-sampling by pyrosequencing (see below).

Overall, our data highlighted a dynamic bacterial population existed in the FASFL, which was directly and closely associated with the water temperature. The FASFL is a complex ecosystem where microflora and aquatic animals coexist and interact. It has been reported that live feed is not a major determinant of microbiota associated with cod larvae (*Gadus morhua*) [30]. Nevertheless, in this study, we could not rule out possible influence of some other environmental variables such as total dissolved solids (TDS) on bacterial community in the niche analyzed, since the TDS values were observed varying as seasonal change as well (data not shown).

### 2.3. Overall Bacterial Community Composition in the FASFL

#### 2.3.1. Bacterial Richness and Diversity

Aquatic bacteria derived from the WHT and WLT were determined by high-throughput 454-pyrosequencing of the 16S rDNA to achieve a detailed phylogenetic-based description of the bacterial compositions. This analysis yielded a total of 18,427 high quality sequences with an average sequence length of 414 bp after the implementation of the quality control criteria as described in Section 3. Of these, 12,802 sequences were retrieved from the WHT, while 5625 sequences were retrieved from the WLT. A large number of operational taxonomic units (OTUs) at the species level were identified using the MOTHUR software, which underlined a highly diverse bacterial community composition in the FASFL analyzed in this study (Table S1).

On the basis of the identified OTUs, bacterial richness was examined by the rarefaction analysis. As shown in Figure S1, the rarefaction curves for the WHT and WLT extended to the horizontal at a phylogenetic distance of 0.20, indicating that almost all of the bacterial diversity at the phyla level was revealed by the pyrosequencing-based analysis, which gave the Good’s coverage of 99.6% and 99.8%, respectively. This result was consistent with the predicted values of OTUs by Chao1 richness calculation (Table S1). However, at phylogenetic distances of 0.05 and 0.03, the identified OTUs derived from the WHT represented 49.0%–54.5% of those estimated by Chao1 analysis (Table S1), implying that more sequencing efforts will be put into this sample in the future to cover the full taxonomic diversity at the genus and species level. Lower bacterial richness assessed by rarefaction analysis than by Chao1 and ACE estimator has also been observed at phylogenetic distances below 5% in previous studies (e.g., [31]). For the WLT, 98.3% and 97.2% coverage of bacterial richness was achieved at the distances below 5%. Moreover, the number of identified OTUs derived from the WLT was *ca*. 6-fold lower than that encountered in the WHT. These results highlighted distinct richness of the bacterial community between the WLT and WHT.

Bacterial diversity of the samples was also assessed by Shannon diversity indices. At the three different phylogenetic distances analyzed in this study, the values of Shannon index ranged from 5.79 to 3.45 for the WHT, which were higher than those encountered in the WLT (4.64 to 3.29) (Table S1). These data revealed a higher degree of bacterial diversity in the WHT when compared to the WLT.

#### 2.3.2. Bacterial Community Composition

**Figure 3:**
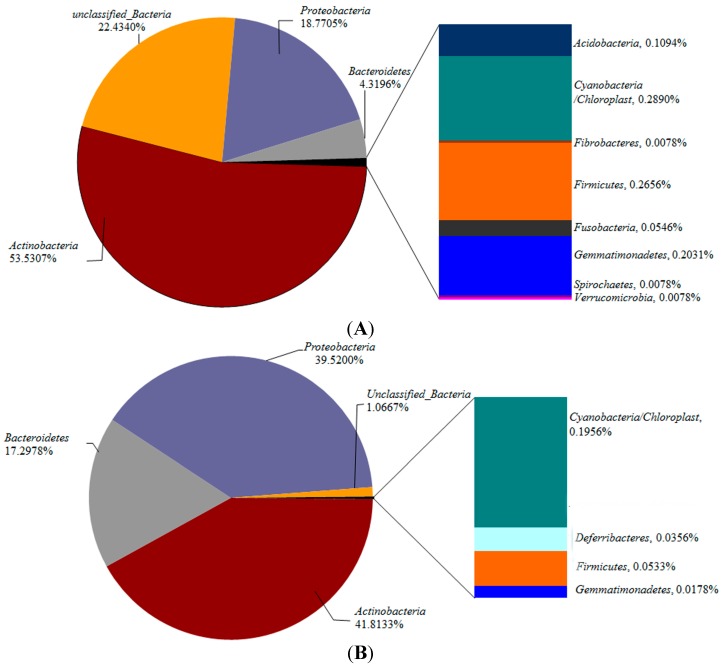
Change in bacterial community composition at the phylum level in the WHT (**A**) and WLT (**B**) samples from the FASFL. Phylum level affiliations of the 454-pyrosequencing sequences retrieved from the samples were performed using the MOTHUR software at a phylogenetic distance of 0.20.

Phylum level affiliations of the sequences retrieved from the samples revealed distinct differences in phylum-level community composition in the FASFL in different seasons. As presented in Figure 3, out of the twelve phyla and candidate phyla identified in this study, the dominant phyla in the bacterial community derived from the WHT were *Actinobacteria*, unclassified bacteria, *Proteobacteria* and *Bacteroidetes*, representing 53.5%, 22.4%, 18.8% and 4.32% of all classified sequences from this sample, respectively. Four phyla were present in lower relative abundance, including *Cyanobacteria/Chloroplast* (0.289%), *Firmicutes* (0.265%), *Gemmatimonadetes* (0.203%) and *Acidobacteria* (0.109%), whereas *Fibrobacteres*, *Fusobacteria*, *Spirochaetes* and *Verrucomicrobia* were found in extremely low abundance at a percentage abundance of 0.0078%, 0.0055% 0.0078%, 0.0078%, respectively. For the WLT, all sequences were affiliated to eight phyla, the dominance of which was *Actinobacteria* (41.80%), *Proteobacteria* (39.52%) and *Bacteroidetes* (17.29%). The lower abundant members included *Cyanobacteria/Chloroplast* (0.195%), *Deferribacteres* (0.04%), *Firmicutes* (0.05%) and *Gemmatimonadetes* (0.017%). Distinct from the WHT, only 1.07% of the sequences retrieved from the WLH fell into the unclassified bacteria group.

Comparison of the phylum-level community composition reinforced the temperature-driven bacterial diversity variation in the aquatic niche analyzed in this study, which lead to three major phylogenetic snapshots. *Actinobacteria* was the most dominant phylum shared between the two samples, followed by *Proteobacteria* and *Bacteroidetes*, which constituted a large proportion of the microflora in the FASFL. These bacteria are also the highest abundant phyla in water samples originated from both aquaculture and freshwater ecosystems, playing important roles in the processes of nutrient cycling and mineralization of organic compounds (e.g., [22,32,33]). Nevertheless, the unclassified bacteria contributed the most to the observed phyla-level diversity variation since *ca*. 21-fold increase in the percentage abundance was observed in the WHT when compared to the WLT. Secondly, three phyla present in lower relative abundance were distributed in the two samples, including *Cyanobacteria/Chloroplast*, *Firmicutes*, *Gemmatimonadetes*, but their relative abundance diminished largely in the WLT. Finally, *Acidobacteria*, *Fibrobacteres*, *Fusobacteria*, *Spirochaetes*,and *Verrucomicrobia* were only found in the WHT, whereas *Deferribacteres* showed an opposite pattern. Due to DGGE sensitivity limitations when dealing with low target abundance samples from natural environments [18], some phyla revealed in this study were not reported in previous DGGE-based studies on aquaculture settings.

**Figure 4:**
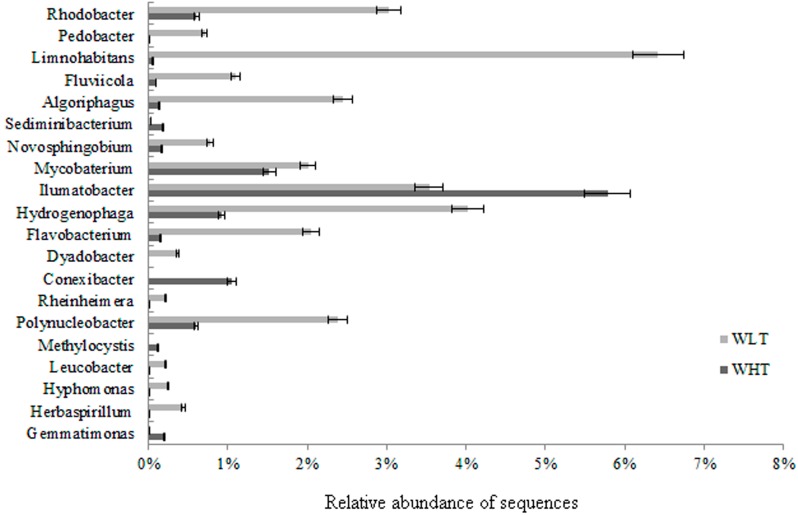
Change in bacterial community composition at the genus level in the WHT and WLT samples from the FASFL. Genus level affiliations of the 454-pyrosequencing sequences retrieved from the samples were performed using the MOTHUR software at a phylogenetic distance of 0.05. The resulting top twenty abundant genera were extracted and their relative abundance was compared.

At the genus level, comparison of the phylogenetic profiles provided further evidence for the temperature-shifted variation in bacterial community composition in the FASFL. As shown in Figure 4, the most abundant genus in the bacterial community derived from the WHT was *Ilumatobacter* representing 5.300% of the classified sequences, followed by *Mycobacterium* (1.523%), *Conexibacter* (1.055%), *Hydrogenophaga* (0.922%), *Rhodobacter* (0.609%) and *Polynucleobacter* (0.601%). *Ilumatobacter* was commonly isolated from the sediments of aquatic habitats (e.g., [34]). The land-based FASFL may serve as an explanation for the observed result. For the WLT, a distinct pattern was revealed, where *Limnohabitans*, *Hydrogenophaga*, *Humatobacter*, *Rhodobacter*, *Algoriphagus*, *Flavobacterium*, *Mycobacterium* and *Fluviicola* were the most responsible for the diversity difference at the genus level, displaying a relative abundance of 6.418%, 4.018%, 3.538%, 3.022%, 2.453%, 2.044%, 2.009% and 1.102%, respectively. These genera comprised *ca*. 23% of the WLT bacterial community. Among the dominant genera shown in Figure 4, *Dyadobacter* was only observed in the WLT, whereas the relative abundance of *Limnohabitans*, *Herbaspirillum*, *Pedobacter*, *Hyphomonas*, *Leucobacter*, *Flavobacterium*, *Algoriphagus* and *Fluviicola* was increased by *ca*. 10–100-fold in the WLT compared to the WHT. However, lower temperature greatly reduced the total number of observed genera, and significantly diminished the relative abundance of a large number of bacteria, such as *Methylocystis*, *Conexibacter*, *Gemmatimonas* and *Sediminibacterium* (Figure 4). In addition, the dominant genera distributed in the two samples were *Ilumatobacter*, *Mycobacterium*, *Hydrogenophaga*, *Rhodobacter* and *Polynucleobacter*, which have also been reported in various aquatic ecosystems. Taken together, our data revealed an extremely diverse community composition with lower relative abundance at the species level in the WHT when contrasted to the phylogenetic profile of the WLT.

**Figure 5:**
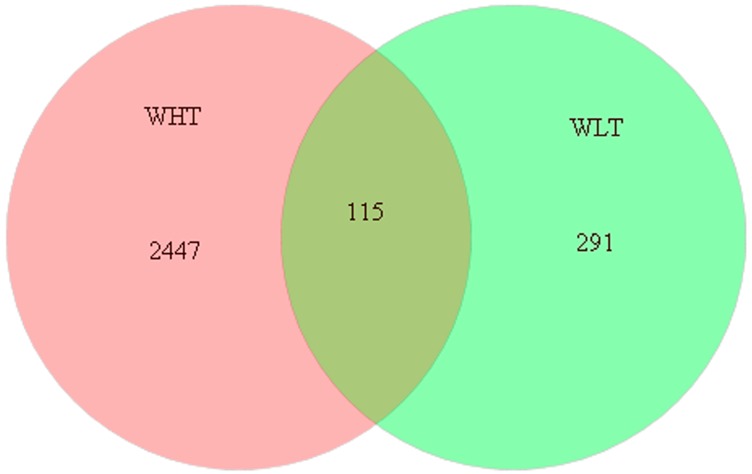
Venn diagram showing the change in bacterial composition at the species level in the WHT and WLT samples from the FASFL. The 454-pyrosequencing sequences retrieved from the samples were analyzed using the MOTHUR software at a phylogenetic distance of 0.03.

Species-level affiliation of the sequences retrieved from the WHT revealed a total of 2562 phylotypes at a phylogenetic distance of 0.03, while 406 phylotypes were identified from the WLT. Of these, a core species containing 115 phylotypes was observed, representing 4.03% of all the richness in the FASFL, regardless of the temperature change (Figure 5).

#### 2.3.3. Potential Pathogenic Bacteria

Base on the pyrosequencing datasets, a search of major bacterial pathogens to aquaculture animals and human revealed different potential risks between the WHT and WLT (Table 2). A total of 25 sequences, representing 0.1357% of all the analyzed sequences, were affiliated closely to seven genus and eleven pathogenetic species that have been reported in the literature [35,36,37]. The following potentially pathogenic species were detected in the WHT sample: *Aeromonas hydrophila*, *Aeromonas caviae*, *Bacillus anthracis*, *Mycobacterium marinum*, *Pseudomonas anguilliseptica*, *Mycobacterium avium* and *Mycobacterium fortuitum*. Among these, *A. caviae, M*. *marinum* and *M*. *avium* were higher temperature-associated and absent from the WLT sample. *A*. *caviae* is one of the most common pathogens related with aquaculture including shrimp, while *M*. *marinum* is also a major pathogen in fish aquaculture. *M*. *avium* exists in various environments including freshwater and can cause human Mycobacterium avium complex disease. Distinct from the WHT, four potential pathogens were detected from the WLT, including *Aeromonas veronii*, *Flavobacterium johnsoniae*, *Serratia marcescens* and *Vibrio cholerae*, all of which are known as human pathogens previously detected in diverse environments in nature. In addition, *A*. *hydrophila*, *B*. *anthracis*, *M*. *fortuitum* and *P*. *anguilliseptica* appeared to more adaptable to temperature change in the FASFL compared to the other pathogens, since they were detected in both WHT and WLT samples. *A*. *hydrophila* is one of the major pathogens in aquaculture, while the other three known as human pathogens contributed the most to the observed potential pathogen enrichment in the FASFL.

**Table 2:** Bacterial pathogens identified in the WHT and WLT samples from the FASFL.

Bacterial Pathogen ^a^	Disease	Source		RAS (%) ^b^	
		Environment	Main Hosts	WHT	WLT
*Aeromonas hydrophila*	Motile aeromonads septicaemia, cholangitis	Freshwater, brackish water, biosolid	Catfish, carp, trout, eel, sturgeon, tilapia, bass	0.0109	0.0054
*Aeromonas caviae*	Speticaemia, gastroenteritis, cholangitis	Freshwater, brackish water, soil, biosolid, agricultural products,	Fish, shrimp, frog, soft-shelled turtle	0.0054	0
*Aeromonas veronii*	Speticaemia, gastroenteritis, cholangitis	Freshwater, biosolid	Human, mosquitos, leeches	0	0.0054
*Bacillus anthracis*	Anthrax	Natural and processed water sources, sewage, biosolid	Human	0.0054	0.0054
*Flavobacterium johnsoniae*	False columnaris	Natural water sources, fish cultures	Barramundi	0	0.0054
*Mycobacterium avium*	Mycobacterium avium complex	Natural water sources, soil, biosolid	Human, farm animals, birds	0.0054	0
*Mycobacterium fortuitum*	Osteomyelitis	River, lake, tap water, soil, dust, biosolid	Human, cattle, frog, other animals	0.0217	0.0163
*Mycobacterium marinum*	Mycobacteriosis	Natural water sources, fish cultures	Atlantic salmo, Seabass, turbot	0.0054	0
*Serratia marcescens*	Conjunctivitis, keratitis, endophthalmitis, tear duct infections	Natural water sources, soil, biosolid	Human, plants, animals	0	0.0109
*Pseudomonas anguilliseptica*	Pseudomonadiasis, Winter disease	Natural water sources, fish cultures	Human, seabream, eel, turbot, ayu	0.0163	0.0109
*Vibrio cholerae*	Vibriosis	Natural water sources, fish cultures, biosolid	Human, croaker fish, puffer fish, grouper, cod, shrimp, big-scale sand smelt, flounder, abalone, seabream, salmon, sweetfish, sheatfish, catfish	0	0.0054

^a^ The bacterial pathogens were reported in literature [35,36,37]; ^b^ Relative abundance of sequence.

## 3. Experimental Section

### 3.1. Sample Collection and Bacterial Genomic DNA Extraction

Water samples were collected from a typical aquaculture farming base for *L*. *vannamei*, located in Fengxian district (N30°51'–E121°23'), Shanghai, China. Each closed and land-based culture pond is about 80 m long and 40 m wide with an average water depth of *ca*. 1.5 m. The ponds are equipped with aerating systems running twice daily to maintain moderate oxygen levels. Water quality was maintained approximately at pH 7.9–8.6 and 0.4‰–2.1‰ salinity during the study period. Water temperature (Wt) in the shrimp ponds is naturally adjusted by the local climate. Sampling was carried out three times monthly from June 2012 to May 2013, except December 2012 to February 2013 when the shrimp aquaculture was annually suspended due to colder weather. Wt was determined using a HACH sensION5 conductivity Meter (HACH Company, Loveland, CO, USA). Surface water was collected at three different sites in each pond using 10 L sterile plastic bottles, and immediately transferred on ice to the laboratory at Shanghai Ocean University in Shanghai, China. Bacterial cells were separated by standard sequential filtration techniques: each water sample was filtered though 8-μm qualitative filter paper to remove large suspended particles, and 1.0 L filtrate was subsequently filtered through polycarbonate membranes with 0.8- and 0.22-μm pore size (47 mm diameter, Millipore, Corcaigh, Ireland), respectively. DNA was extracted from three filters of each sample using the QIAamp DNA Stool Mini Kit (QIAGEN Biotech Co. Ltd., Hilden, Germany) according to the manufacturer’s instruction. The concentration of DNA in the samples was determined using a multi-mode microplate reader BioTek Synergy™ 2 (BioTek Instruments, Inc., Winooski, VT, USA).

### 3.2. PCR-DGGE and Data Analysis

The V3 variable regions of bacterial 16S rRNA genes were amplified by PCR using the primer pair P3 (5'-GCCCGCCGCGCGCGGCGGGCGGGGCGGGGGCACGGGGGGCCTACGGGAGGCAGCAG-3') and P2 (5'-ATTACCGCGGCTGCTGG-3') as described previously [38]. The expected length of amplified PCR products is 193 bp with a GC clamp attached to their 5'-termini [38]. Oligonucleotide primers were synthesized by Shanghai Sangon Biological Engineering Technology and Services Co., Ltd. (Shanghai, China). The PCR amplification was performed in a 20 μL reaction volume as described previously [39]. Amplification was performed in a MastercyclerW pro PCR thermal cycler (Eppendorf, Hamburg, Germany) with a touch down PCR protocol [38]. A sample (5 μL) of each PCR product was analyzed by agarose gel electrophoresis with a 1.5% agarose gel. Amplified DNA fragments were visualized and imaged by Molecular Imager^®^ Gel Doc™ XR + System (Bio-Rad Laboratory, Hercules, CA, USA).

The vertical DGGE was performed using a DCode™ Universal Mutation Detection System (Bio-Rad, Hercules, CA, USA) according to the manufacturer’s instructions. Each sample (400–500 ng) combined from three independent amplicons was loaded per well onto a 8% polyacrylamide gel (acrylamide:bis-acrylamide, 37.5:1) with a liner denaturing gradient range of 45%–55% formed with 7 M urea and 40% (*v*/*v*) deionized formamide. The denaturing gradient gel was run at 60 V for 16 h with a constant temperature of 60 °C in 1× TAE buffer (40 mM Tris base, 20 mM acetic acid, 1.0 mM Na_2_-EDTA, pH 8.0). Following electrophoresis, the gel was stained three times with 6 mL of 0.01% SYBR^®^ Green I Nucleic Acid Gel Stains (Invitrogen, Carlsbad, CA, USA) for 10 min according to the manufacturer’s instructions, rinsed and imaged by Molecular Imager^®^ Gel Doc™ XR System (Bio-Rad, Hercules, CA, USA). The DGGE profiles were analyzed using the Quantity One^®^ 1-D Analysis software (version 4.6.2; Bio-Rad, Hercules, CA, USA). DNA banding patterns were compared using pairwise similarity matrices calculated with the Dice’s coefficient [27], and cluster analysis was performed using the unweighted pair group method with arithmetic mean (UPGMA) via the software. The PRIMER (Version 5.2.8, PRIMER-E Ltd., Ivybridge, Devon, UK) was used for analysis of similarity (ANOSIM) [40]. The statistical significance of differences in microbial community composition was determined by IBM SPSS Statistics (Version 19) with one-way ANOVA (IBM, Armonk, NY, USA). The RDA analysis was performed using the CANOCO Version 4.5 [41].

### 3.3. Sequencing and Phylogenetic Analysis

Distinct DNA bands on the DGGE gels were individually excised and re-amplified using the primer pair 341f (5'-CCTACGGGAGGCAGCAG-3') (without GC clump) and 534r (5'-ATTACCGCGGCTGCTGG-3') according to the method described previously [38,42]. The PCR product was purified using AxyPrep DNA Gel Extraction Kit (Axygen, Union City, CA, USA), and ligated into the pGM-T cloning vector (TianGen Biotech Co., Ltd., Beijing, China) according to the method described previously [43]. Ligated DNA transformation and positive colony identification were performed as described previously [44]. The *E*. *coli* TOP10 (genotype: F^−^*mcr*A∆ (*mrr*-*hsd*RMS-*mcr*BC) ψ80 *lac*Z∆M15∆*lac*X74 *rec*A1 *ara*D139∆ (*ara-leu*) 7697 *gal*U *gal*K *rps*L(Str^r^) *end*A1 *nup*G), (TianGen Biotech Co., Ltd., Beijing, China) was used as a host strain for DNA cloning. Plasmid DNA was prepared using the MiniBEST Plasmid DNA Extraction Kit Ver.2.0 (Japan TaKaRa BIO, Dalian Company, Dalian, China).

Automated DNA sequencing was carried out using ABI 3730xl capillary sequencer (Applied Biosystems, Foster City, CA, USA) and BigDye^®^ terminator Version 3.1 cycle sequencing kit (Perkin-Elmer, Maltham, MA, USA) at the China Human Genome Centre (Shanghai, China). Sequencing reads were checked for chimera formation with the Ribosomal Database Project (RDP, http://rdp.cme.msu.edu/), and inferred for the closest relative using the Basic Local Alignment Search Tool (BLAST) (http://www.ncbi.nlm.nih.gov/BLAST). The sequence data was deposited in the NCBI Sequence Read Archive under SAMN02698684-97.

### 3.4. Pyrosequencing and Data Analysis

Pyrosequencing was carried out using the 454/Roche GS-FLX Plus System (Roche Diagnostics, Basel, Switzerland) at the Hanyu Biotech Co., Ltd., the China Human Genome Centre (Shanghai, China), according to the standard protocols of the manufacturer. The V3–V4 variable regions of bacterial 16S rRNA genes were amplified by PCR using the universal bacterial primers with an 8-bp barcode and a GS-FLX sequencing adaptor (not shown). The MOTHUR Ver.1.32.0 software was employed for most of the sequence processing and analyses [45]. Raw sequence data were pre-processed to remove primers and barcodes, poor quality reads (>1% sequencing error rate), and the reads less than 200 bp in length. Chimeric reads were detected using the UCHIME software [46]. Silva/Greengenes database was used to remove non-targeted sequence contamination [47]. Qualified sequences were clustered into OTUs in MOTHUR at 0.03, 0.05 and 0.20 phylogenetic distance threshold, defined at species, genus and phylum level, respectively [44]. For read level taxonomic analysis, the RDP Classifier 2.2 of the RDP was used at a confidence threshold value of 80% [48]. Bacterial Chao1 richness [49], Shannon diversity index [50], Good’s coverage, rarefaction curves, Venn diagram were calculated and created using the MOTHUR based on the OTUs clustering data. The sequence data was deposited in the NCBI Sequence Read Archive under the SAMN02699090-91.

## 4. Conclusions

This study constitutes the first investigation of temperature influence on bacterial community composition in freshwater aquaculture system farming of *Litopenaeus vannamei*. Water samples were collected over one year period, and aquatic bacteria were characterized by PCR-DGGE and pyrosequencing of the 16S rDNA. The results revealed a specific and dynamic bacterial population structure with considerable variation over the seasonal change. Phylogenetic analysis of the 18,427 sequences substantiated that the WHT harbored remarkably higher diversity and richness in bacterial composition when compared to the WLT. A large number of unclassified bacteria contributed the most to the observed variation in the system, albeit *Actinobacteria*, *Proteobacteria* and *Bacteroidetes* constituted a major proportion of the microflora in the FASFL. Three phyla present in lower relative abundance were distributed in WHT and WLT, including *Cyanobacteria/Chloroplast*, *Firmicutes* and *Gemmatimonadetes*. In addition, *Acidobacteria*, *Fibrobacteres*, *Fusobacteria*, *Spirochaetes* and *Verrucomicrobia* were only found in the WHT in extremely low abundance, whereas *Deferribacteres* showed an opposite pattern. At the genus level, phylogenetic analysis also revealed distinct profiles: *Ilumatobacter*, *Mycobacterium*, *Conexibacter*, *Hydrogenophaga*, *Rhodobacter* and *Polynucleobacter* were the highest abundant genus in the WHT, whereas *Limnohabitans*, *Hydrogenophaga*, *Humatobacter*, *Rhodobacter*, *Algoriphagus*, *Flavobacterium*, *Mycobacterium* and *Fluviicola* were the most responsible for the diversity difference. At the species level, a total of 2562 phylotypes were identified in the WHT, while 406 phylotypes were found in the WLT. A core species containing 115 phylotypes was observed, representing 4.18% of all the richness in the FASFL. Eleven potential pathogenetic species were identified with different phylogenetic patterns in the WHT and WLT. It will be interesting to characterize these bacteria in future research. Our data demonstrated a temperature-driven substantial shift in bacterial population in the FASFL. The results provide guidance for aquatic animal disease control in shrimp aquaculture, and also constitute an important step in extending our knowledge of microbial ecology in aquaculture ecosystem, particularly waterborne pathogenic bacteria mediated by climate change.

## Supplementary Files

Supplementary File 1
