# Supplementary material for: Identification of Bacterial Community Composition in Freshwater Aquaculture System Farming of Litopenaeus vannamei Reveals Distinct Temperature-Driven Patterns
Source: Int J Mol Sci. 2014 Aug 7;15(8):13663–80. doi: 10.3390/ijms150813663 (PMC4159818; doi:10.3390/ijms150813663)
Supplement: Supplementary File 1 [file ijms-15-13663-s001.pdf]

Supplementary Information

Figure S1. Rarefaction analysis of the 454-pyrosequencing data retrieved from the WHT and WLT samples in the FASFL. The operational taxonomic units (OTUs) were analyzed at 0.20, 0.05 and 0.03 phylogenetic distances, respectively.

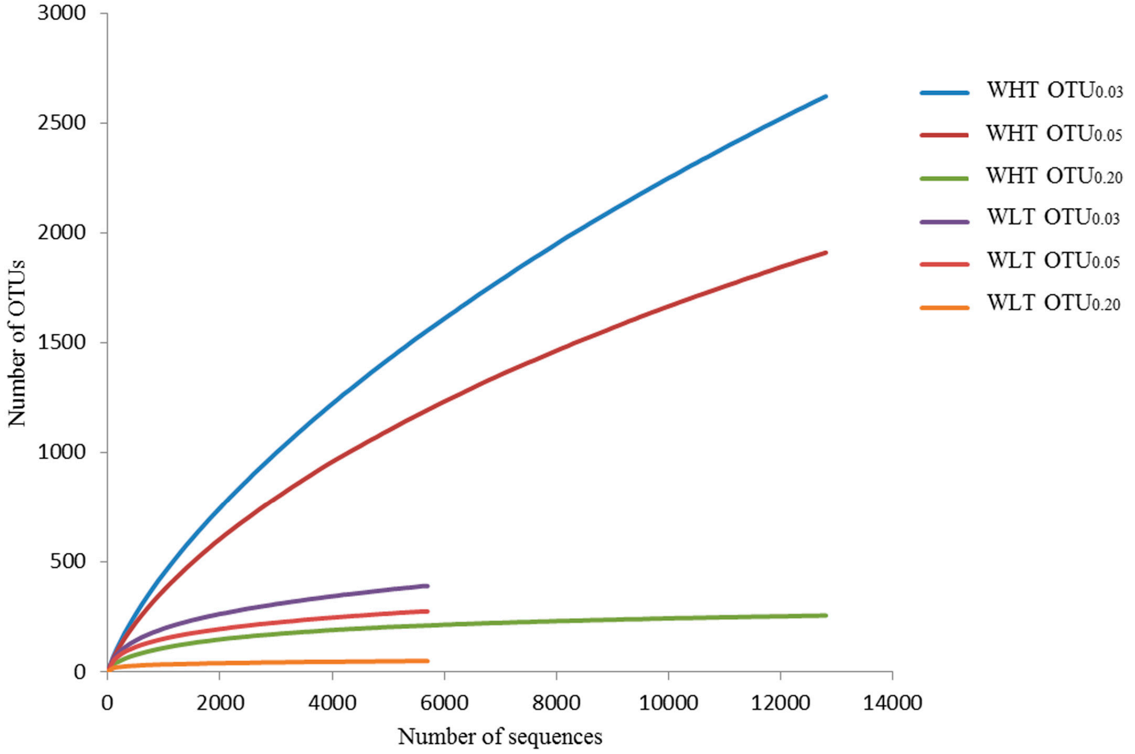

Table S1. Richness and diversity of bacterial communities derived from the WHT and WLT samples in the FASFL.

Sample	Cutoff	OTUs	Chao1	Shannon Index	Good's Coverage
WHT	0.03	2562	5,229	5.79	0.879
	0.05	1892	3473	5.44	0.920
	0.20	256	297	3.45	0.996
WLT	0.03	406	646	4.64	0.972
	0.05	301	402	4.34	0.983
	0.20	49	58	2.39	0.998
